# High out‑of‑pocket spending and financial hardship at the end of life among cancer survivors and their families

**DOI:** 10.1186/s13584-023-00572-x

**Published:** 2023-07-06

**Authors:** Jingxuan Zhao, K. Robin Yabroff

**Affiliations:** Surveillance and Health Equity Science, American Cancer Society, 3380 Chastain Meadows Pkwy NW Suite 200, Kennesaw, GA 30144 USA

**Keywords:** Cancer, End-of-life care, Financial hardship

## Abstract

Cancer is one of the most expensive medical conditions to treat worldwide, affecting national and local spending, as well as household budgets for patients and their families. In this commentary about a recent paper from Tur‑Sinai et al., we discuss the high out-of-pocket spending and medical and non-medical financial hardship faced by cancer patients and their families at the end-of-life in Israel. We provide recent information about the costs of health care in Israel and other high-income countries with (i.e., Canada, Australia, Japan, and Italy) and without universal health insurance coverage (i.e., United States, a country with high healthcare costs and uninsurance rate), and highlight the role of improving health insurance coverage and benefit design in reducing financial hardship among cancer patients and their families. Recognizing that financial hardship at the end of life affects both patients and their families, developing comprehensive programs and policies in Israel as well as in other countries is warranted.

## Background

Cancer is one of the most expensive medical conditions to treat worldwide [1, 2], affecting national and local spending, as well as household budgets for patients and families. Historically, cancer patients and survivors have higher out-of-pocket spending compared to similar individuals without a cancer history [3, 4]. People with cancer and their families are also more likely to experience medical and non-medical financial hardship, including difficulty paying medical bills and medical debts; distress about paying medical bills; delaying or forgoing medical care and prescription drugs due to costs [5–7]; food insecurity; and worry about rent, mortgage, and other housing costs [6, 8]. Advances in expensive cancer therapies, such as immunotherapies and targeted therapies [9, 10], and greater treatment duration and intensity [11, 12], result in even greater costs of cancer care, especially at the end-of-life [13].

Understanding out‑of‑pocket spending and financial hardship among cancer patients and their informal caregivers at the end-of-life in Israel is the focus of a recent study by Tur‑Sinai et al. [14]. The authors surveyed 491 first-degree relatives of cancer patients who died 3–6 months before the interview in multiple medical centers in Jerusalem during 2018–2019. In the last 6 months of patients’ life, 84% had out‑of‑pocket spending, with an average of $5800 on medicines, $8000 on private professional caregivers, and $2800 on private nurses. A large percentage of families also reported indirect health care spending, such as spending for traveling to medical centers (70%) and food away from home while receiving care (60%). They also found greater financial hardship due to high out-of-pocket spending if patients were unable to remain alone, if they did not have supplemental private health insurance coverage, or if they had lower income or lower educational attainment compared to their counterparts.

Cancer patients often face intensive care at the end of life, which can translate into high medical costs. An earlier study conducted in Israel found that end-of-life care costs during the last 12 months of patients’ life was about 20 times higher than the cost for other phases of care; and the end-of-life care cost for cancer patients were about 50% higher than decedents with other health conditions [15]. Another study conducted in the United States (US) estimated that cancer patients aged ≥ 65 years with Medicare coverage had net annualized out-of-pocket spending for medical services and prescription drugs of $4271, $2443, and $593 (in 2019 US dollars), at the end-of-life/last 12 months of life, initial 12 months following diagnosis, and continuing (period between initial and end-of-life phases), phases of care, respectively, compared to similar individuals without a cancer history [4]. This high out-of-pocket spending at the end-of-life likely imposes financial burden among families. In another study, about a third of US household experiencing cancer deaths spent most or all of their savings on patient end-of-life care, even though most of these patients were ≥ 65 years and covered by Medicare [16]. Therefore, understanding the high out-of-pocket spending at the end of life for cancer patients and identifying modifiable risk factors for financial hardship is critical for improving family outcomes.

As shown by Tur‑Sinai et al., most cancer patients and their families also incurred nonmedical spending, such as travel and food, as part of end-of-life care, which can also impose financial burdens. Earlier studies showed that having expenses on travel and food when seeking care was common in the last few months of cancer patients’ life in multiple countries, including the US [17], Canada [18], and England [19]. Some programs have been developed to help cancer patients with travel expenses for treatment. For example, the Israel Lemonade Fund provides free rides to chemotherapy and radiation treatments [20]; the American Cancer Society offers free rides to treatment through the Road to Recovery program [21], and free lodging during treatment through the Hope Lodge program to cancer patients and their families [22]. Helping cancer patients and their families navigate their options and eligibility for these assistance programs is an important component of ensuring access to cancer care.

In Israel, where the study by Tur‑Sinai et al. was based, universal health insurance is provided to all residents under the National Health Insurance (NHI) Law since 1995. The NHI offers comprehensive coverage of oncology drugs and no co-payments are charged for these drugs; but patients may incur out-of-pocket spending for services such as specialist visits and prescription drugs that are not included in the "National List of Health Services" in Israel [23]. Almost all individuals in Israel purchase private health insurance plans through NHI or commercial health insurance coverage as a supplement to the public coverage offered by NHI [23]. As noted by Tur‑Sinai et al., lack of supplemental private health insurance coverage was associated higher odds of having financial hardship among cancer patients and their families.

**Fig. 1:**
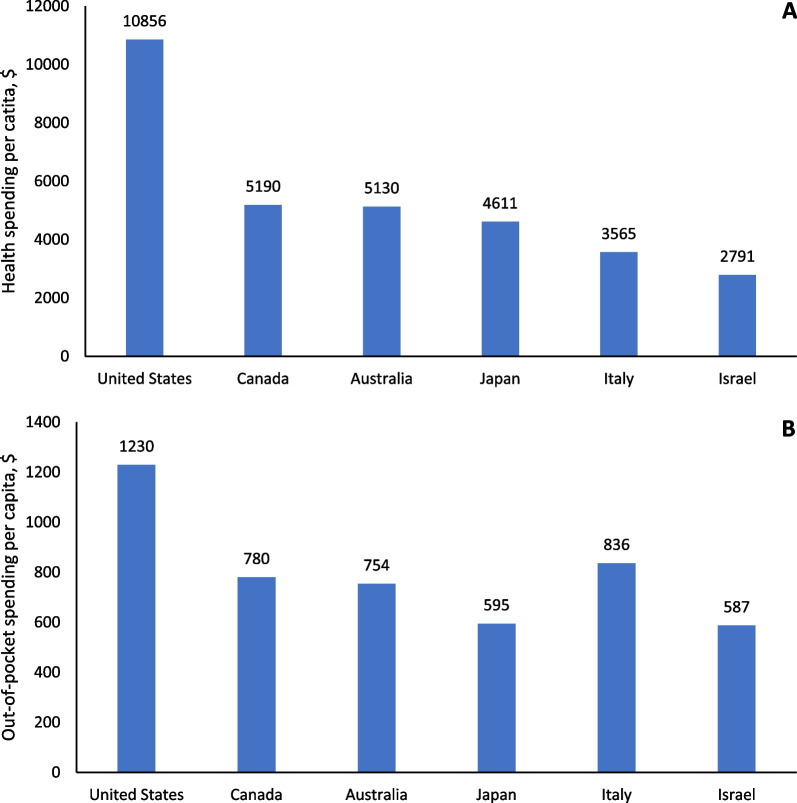
Total health spending per capita (**A**) and out-of-pocket spending per capita (**B**) by country, 2019. Data source: OECD: Organization for Economic Co-operation and Development (OECD) repository, https://www.oecd-ilibrary.org/docserver/ae3016b9-en.pdf?expires=1680289022&id=id&accname=guest&checksum=52BADFE1226DC51008F04A7F8087A289

Few studies have compared health care spending in Israel with other countries; most research related to patient out-of-pocket spending and financial hardship has been conducted in the US [24–26]. In contrast to countries like Israel with universal health insurance coverage, the US does not have universal coverage and millions of people are uninsured [27]. Healthcare costs in the US also rank among the most expensive worldwide [28]. As shown in Fig. 1, the total health spending per capita in 2019 was $10,856 and patient out-of- pocket spending was $1230 in the US, much higher than the corresponding spending in Israel ($2791 and $587, respectively) and other high income countries [29]. When compared to other high income countries listed in Fig. 1, such as Canada, Australia, and Japan, where a universal health insurance coverage is provided, Israel had the lowest total health spending per capita and patient out-of- pocket spending; however, cancer patients are still at risk for financial hardship.

Improving health insurance coverage and benefit design is a focus for reducing financial hardship among cancer patients and their families. In the US, several state and federal programs, such as Medicaid expansion and health insurance Marketplaces were established and implemented under the Affordable Care Act (ACA) to improve health insurance coverage in the US, which are associated with lower risks care affordability problems among cancer survivors [30, 31]. However, financial hardship remains common among cancer survivors in the US after the ACA [5], indicating that improving insurance coverage might be necessary, but not sufficient to mitigate the financial burden from cancer and its treatment, as some services, such as professional caregivers and certain medicines might not be covered or adequately covered by health insurance. As shown by Tur‑Sinai et al., 42% patients had out-of-pocket spending for medicine and 32% had out-of-pocket spending for professional caregivers, even though all patients had insurance coverage through the NHI and a majority of them purchased supplement commercial health insurance. Therefore, modifying existing benefits and reducing cost sharing for these services may relieve the financial burden among cancer survivors and their families. In addition, patients with cancer may have health insurance literacy problems, which means they might not be able to choose the best plans for their needs or might not be aware of all the benefits provided by their plans. Limited health insurance literacy is associated with financial hardship among cancer survivors [32, 33]. Tur‑Sinai et al. also highlighted the important role for health care providers and their teams in helping patients and their families navigate their entitlements under the NHI that could potentially reduce their cost sharing and out-of-pocket spending.

Another important finding of the study by Tur‑Sinai et al. is that financial hardship related to end-of-life cancer care affects both patients and their families. Beside bearing the financial burdens due to the high cost of cancer care, family members often act as caregivers throughout patients’ treatment trajectory [34–36]. For family caregivers who also work for pay, taking care of patients may result in more missing work hours, less productivity, lower pay, or even loss of employer-sponsored health insurance coverage or paid sick leave benefits, which imposes greater financial hardship for the family [35]. Therefore, it is important for employers to provide accommodations for caregivers as well.

## Conclusions

The high out-of-pocket spending may impose medical and non-medical financial hardship on cancer patients and their families at the end-of-life. Improving health insurance coverage and benefit design may reduce their financial hardship. With greater attention to understanding different aspects of financial hardship faced by cancer survivors and their families, the study by Tur-Sinai et al. provides important insights to development of comprehensive programs and policies in Israel as well as in other countries.

## Data Availability

The datasets analyzed during the current study are available in the OECD repository, https://www.oecd-ilibrary.org/docserver/ae3016b9-en.pdf?expires=1680289022&id=id&accname=guest&checksum=52BADFE1226DC51008F04A7F8087A289.

## Abbreviations

US: United States
NHI: National Health Insurance
ACA: Affordable Care Act
